# Feasibility analysis and efficacy evaluation of computed tomography–guided microwave ablation combined with radioactive ^125^I particles in the treatment of lung nodules

**DOI:** 10.20452/wiitm.2025.17936

**Published:** 2025-03-24

**Authors:** Yu-Qing Shan, Fan-Xia Lin, Li Wang, Hai-Yu Wang

**Affiliations:** Department of Medical Imaging Centre, The People’s Hospital of Rizhao, Rizhao, China

**Keywords:** lung cancer, microwave ablation, radioactive ^125^I particles

## Abstract

**INTRODUCTION:**

Lung cancer is one of the leading causes of mortality worldwide and it requires early detection and treatment. Microwave ablation (MWA), a minimally invasive technique, is effective in early stages of lung cancer, but may cause complications when applied near sensitive structures. Such cases require the use of ^125^I seed implantation. Our study retrospectively analyzed 58 lung nodule patients treated with MWA in combination with simultaneous or delayed radioactive particle implantation to evaluate the impact of implant radiation therapy on patient outcomes and complication rates.

**AIM:**

The aim of this study was to evaluate the feasibility of MWA combined with ^125^I seed implantation for treating ipsilateral single pulmonary nodules.

**MATERIALS AND METHODS:**

This retrospective study included 58 patients who underwent computed tomography (CT)-guided percutaneous pulmonary biopsy of ipsilateral single nodules combined with MWA and simultaneous or subsequent radioactive particle implantation. The experimental group (n = 28) received both treatments at the same time, while in the control group (n = 30), ^125^I seed implantation was performed 1 week after MWA or after resolution of complications. Data on clinical outcomes, complications, and chest CT findings at 1, 3, and 6 months after the procedure were collected.

**RESULTS:**

All procedures were completed with a 100% technical success rate. Complication rates (hemoptysis, pneumothorax, pleural effusion) were similar in both groups (*P* = 0.76, *P* = 0.8, *P* = 0.7, respectively), whereas duration of hospitalization was longer in the control group (*P* = 0.03). At 3 months, the total effective rate was 70% in the control group and 75% in the experimental group (*P* = 0.67), with 100% local control in both groups. At 6 months, the total effective rate was 80% and 82.1%, respectively (*P* = 0.84), with continued 100% local control.

**CONCLUSIONS:**

Combining CT-guided biopsy with immediate MWA and particle implantation for ipsilateral single pulmonary nodules does not increase the incidence of complications. Specimens are suitable for clinical and pathological testing, and lesion control rates meet clinical standards. This combined approach is feasible and safe.

## INTRODUCTION

Despite lung cancer ranking second in incidence globally, it remains one of the leading causes of mortality, which is why early detection, diagnosis, and treatment are crucial in treating it.^1^ Local thermal ablation, a precise and minimally invasive technique, has been increasingly employed in the treatment of early lung cancer with the annual number of treated cases rapidly rising.^2^^,^^3^ This technique boasts minimal trauma, clear efficacy, high safety, strong reproducibility, and broad applicability across different patient populations. However, microwave ablation (MWA) of nodules adjacent to the pleura and pericardium may lead to complications, such as vagal nerve reactions and massive bleeding. This therapy achieves ultrahigh radiation doses within the tumor target area, while the dose around the target rapidly diminishes resulting in minimal exposure of normal tissues and surrounding organs.^4^ Therefore, ^125^I seed implantation has gained widespread recognition as an effective treatment for lung nodules. In this study, we retrospectively analyzed the occurrence of postoperative complications and short-term efficacy at 1-, 3-, and 6-month follow-ups in 58 patients with lung nodules who underwent MWA therapy either simultaneously or selectively combined with particle implantation therapy.

## AIM

The aim of this study was to evaluate the feasibility of MWA combined with particle implantation for treating ipsilateral single pulmonary nodules.

## MATERIALS AND METHODS

### Patients and procedure

A retrospective analysis was conducted on the clinical and follow-up data of 58 patients with lung nodules who underwent percutaneous lung puncture thermal ablation, either simultaneously or selectively combined with particle implantation at the Medical Imaging Centre of Rizhao People’s Hospital between January 2022 and December 2024. Among these patients, 37 were men and 21 were women aged from 28 years to 76 years. All of them had previously undergone lung biopsy confirming their lung nodule lesions as malignant. A randomization method was used to divide the patients into 2 groups: an MWA combined with particle implantation group (experimental group) which included 28 participants and an MWA selective particle implantation group (control group) which consisted of 30 participants.

Inclusion criteria concerned patients meeting the following conditions: 1) maximum lesion diameter of ≤3 cm; 2) inability to tolerate general anesthesia or surgical intervention; 3) infeasibility of surgical resection due to the lesion location or limited lung function reserve; 4) no possibility of complete resection of multiple lesions; and 5) shrinking or stabilization of the lesion after treatment with other methods, necessitating ablation or particle therapy to consolidate the therapeutic effect.

### Instruments and methods

Chest scanning was performed using a Siemens SOMATOM go.Up series 64-slice spiral computed tomography (CT) machine (Siemens, Erlangen, Germany) with the following parameters: tube voltage of 120 kV, tube current of 285 mAs, slice thickness of 5 mm, and reconstructed slice thickness of 2 mm. The ablation equipment utilized was an ECO-100A microwave ablation therapeutic device (Nanjing Yigao Microwave System Engineering Co., Ltd., Nanjing, China) operating at a frequency of 2450 MHz with an adjustable output power range of 0 to 50 W. The ECO 18G microwave ablation needle model ECO-100 AL5 (Nanjing Yigao) had specifications of 1.6 mm × 150 mm. The patient’s position was determined based on the predefined puncture path, and local anesthesia was administered using 1% lidocaine at the puncture site.

In the experimental group, preoperative assessments were undertaken to establish the patient’s positioning, the strategy for nodule management, puncture route, and other pertinent variables. After the routine chest CT scan, the skin puncture entry point was defined, the adjacent area was sanitized, and anesthesia was administered to several sites using 10 milliliters of 1% lidocaine. Guided by CT imaging, both the ablation probe and the particle injector were inserted percutaneously into the precise location of the lesion. After verification via another CT scan, multiple nodules were targeted for ablation and particle seeding. The ablation power was calibrated at 40–60 W, with a total ablation time of 4 to 5 minutes, yielding a maximum ablation zone of 3.5 cm by 4 cm. Upon completion of ablation and withdrawal of the probe, CT imaging demonstrated elevated density of lung tissue surrounding the lesion, creating a halo of 5 to 10 mm outside the lesion borders. The radiation oncologist formulated a treatment protocol with a target volume dose spanning from 100 to 140 Gy. With CT imaging as a guide, once the implantation needle precisely accessed the tumor site, ^125^I particles (Saili, Tianjin, China) with a dose of 0.7 mCi were administered at intervals of 1 to 1.5 cm. A follow-up chest CT scan was performed to test for any potential complications, such as pneumothorax or hemorrhage.

The preoperative preparation of the control group was identical to that of the experimental group. Under CT guidance, the ablation needle was percutaneously inserted into the location of the lesion. The ablation parameters utilized were consistent with those employed in the experimental group. Upon cessation of ablation and subsequent removal of the needle, CT scanning revealed increased density of lung tissue surrounding the lesion, forming a halo that surpassed the lesion boundaries by 5–10 mm. The ablation range was then evaluated. Particle implantation was conducted either 1 week after MWA or once the complications had fully resolved. The preoperative procedure for particle implantation was the same as described above, and the particle specifications and administration methods were analogous to those used in the experimental group. After completion, the particle needle was removed. A subsequent chest CT scan was performed to check for any complications, such as pneumothorax or bleeding.

### Efficacy and complications

Promptly after the procedure, a chest CT scan was performed to evaluate the ablation zone, which was deemed adequate if a ground-glass opacity with a thickness ranging from 0.5 to 1 cm was visible on the periphery of the nodule.^5^ Furthermore, a postoperative assessment of the treatment volume covered by particle radiotherapy was performed to check whether it aligned with the anticipated outcomes. Technical success rates were calculated accordingly.

Complications including pneumothorax, pleural effusion, and hemoptysis, were evaluated within 24 hours after surgery in accordance with the guidelines established by the International Collaborative Group on Tumor Ablation.^6^ The postoperative complications were assessed using the following criteria: pleural effusion was categorized as minor (<500 ml), moderate (500–1000 ml), or significant (>1000 ml); pneumothorax was categorized as mild (<20% lung compression), moderate (20%–50% lung compression), or severe (>50% lung compression); and hemoptysis was categorized as slight (<10 ml), moderate (10–100 ml), or substantial (>100 ml).

### Follow-up

Postoperative follow-up was conducted for a period of 6 months to monitor for complications such as needle tract metastasis, pulmonary embolism, bronchopleural fistula, or mortality. The follow-up included plain and contrast-enhanced chest CT scans at 1, 3, and 6 months postoperatively. The diameter of the nodular enhancement area in the arterial phase was measured during these scans. All sample size data were obtained from contrast-enhanced CT images performed before and after treatment, and the maximum diameter line of the lesion in the same plane was selected for comparison. Data collected 1 month after the surgery were used as baseline for evaluating efficacy at 3 and 6 months postoperatively. The efficacy was assessed using several criteria. Complete response (CR) was defined as a reduction in the ablation target area or the formation of a scar, accompanied by a ≥50% decrease in the diameter of the implanted particle nodule and no enhancement of the lesion. Partial response (PR) was defined as the formation of honeycomb or cavity structures within the lesion after ablation and particle implantation, with a ≥30% decrease in the diameter of the enhancement area in the arterial phase. Stable disease (SD) was defined as a decrease in lesion density with either a <30% decrease or <20% increase in the diameter of the enhancement area in the arterial phase. Progressive disease (PD) was defined as a ≥20% increase in the diameter of the enhancement area in the arterial phase or the emergence of new lesions. The overall response rate was calculated as CR + PR/total number of cases × 100%, and the local control rate was determined as CR + PR + SD/total number of cases × 100%.^7^ Additionally, the total duration of hospitalization, from the date of surgery to the date of discharge, was calculated.

### Statistical analysis

Statistical analysis was conducted using SPSS software, version 26.0 (SPSS Inc., Chicago, Illinois, United States). Quantitative data that followed a normal distribution were presented as mean (SD), and intergroup comparisons were performed using the independent sample *t* tests. Comparisons of the patients’ sex, smoking history, complications, and treatment outcomes between the groups were conducted using the χ^2^ test. A *P* value of less than 0.05 was considered significant.

### Ethics

This study received approval from the local institutional review board (KYSQ014-2024), and all patients provided written informed consent to participate in it. The study complies with the ethical guidelines outlined in the Declaration of Helsinki, originally established in 1975 and revised in 2000.

## RESULTS

### Basic information

No significant differences were observed between the 2 groups in terms of sex, age, body mass index, smoking history, pathological type, and Tumor, Node, Metastasis classification. Detailed information is presented in TABLE 1.

**TABLE 1:** Clinical characteristics of the patients and their lesions

Parameter		Control group (n = 30)	Experimental group (n = 28)	*P* value
Age, y, mean (SD)		66.33 (6.42)	64.39 (7.17)	0.89
Sex	Men	18 (60)	19 (67.9)	0.53
	Women	2 (40)	9 (32.1)	
BMI, kg/m^2^, mean (SD)		23.80 (3.1)	23.99 (2.82)	0.62
Smoker		8 (26.7)	9 (32.1)	0.19
Pathological type	Squamous cell carcinoma	21 (70)	9 (30)	0.46
	Adenocarcinoma	17 (60.7)	11 (39.3)	
TNM staging	Phase III	26 (86.7)	4 (13.3)	0.26
	Phase IV	21 (75)	7 (25)	

Data are presented as number (percentage) of patients unless indicated otherwise.

Abbreviations: BMI, body mass index; TNM, Tumor, Node, Metastasis

### Complications and hospital stay

All the patients underwent the procedure successfully, achieving a technical success rate of 100% (58/58). In the experimental group, 3 patients experienced hemoptysis (3/28; 10.71%), 4 had small pneumothorax (4/28; 14.28%), and 2 had pleural effusion (2/28; 7.14%). In the control group, 4 patients experienced hemoptysis (4/30; 13.33%), 5 had small pneumothorax (5/30; 16.67%), and 3 had pleural effusion (3/30; 10%). No differences were observed between the 2 groups in the incidence of pneumothorax, pleural effusion, or hemoptysis. However, there was a notable discrepancy in the duration of hospital stay which was 4.54 (1.29) days for the experimental group and 7.97 (0.93) days for the control group (*P* = 0.03). Detailed information is shown in TABLE 2.

**TABLE 2:** Complications within 24 hours of surgery in the 2 groups

Complications	Control group (n = 30)	Experimental group (n = 28)	*P* value
Pneumothorax	5 (16.67)	4 (14.28)	0.8
Pleural effusion	3 (10)	2 (7.14)	0.7
Hemoptysis	4 (13.33)	3 (10.71)	0.76
Duration of hospitalization, d, mean (SD)	7.97 (0.93)	4.54 (1.29)	0.03

Data are presented as number (percentage) of patients unless indicated otherwise.

### Efficacy

**FIGURE 1:**
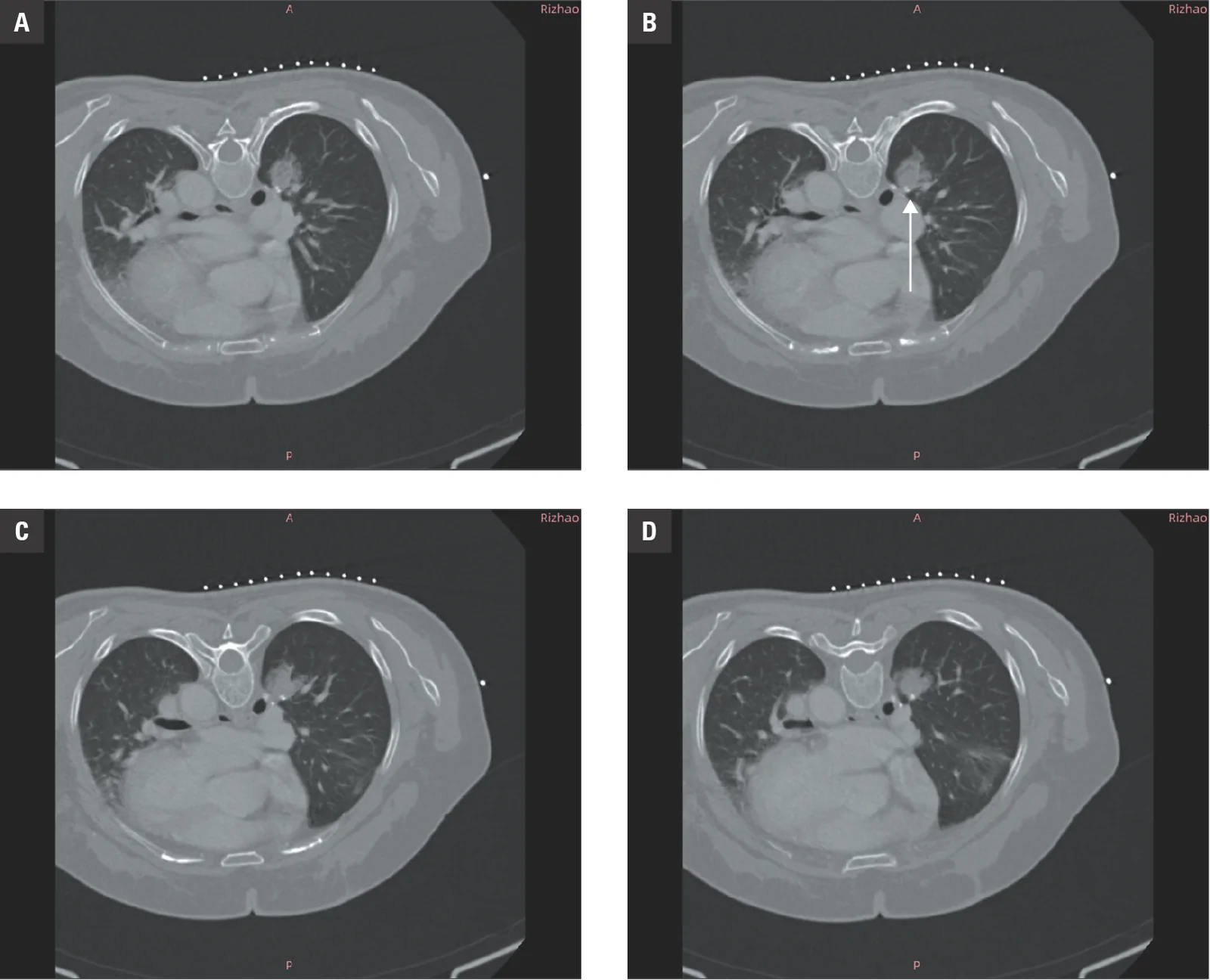
**A**–**D** – imaging data of a 64-year-old woman who underwent surgery on the lower lobe of her right lung. The arrow (**B**) indicates the shadow of the surgical stapler after the operation. The local soft tissue mass was confirmed by biopsy to be a local tumor recurrence.

**FIGURE 2:**
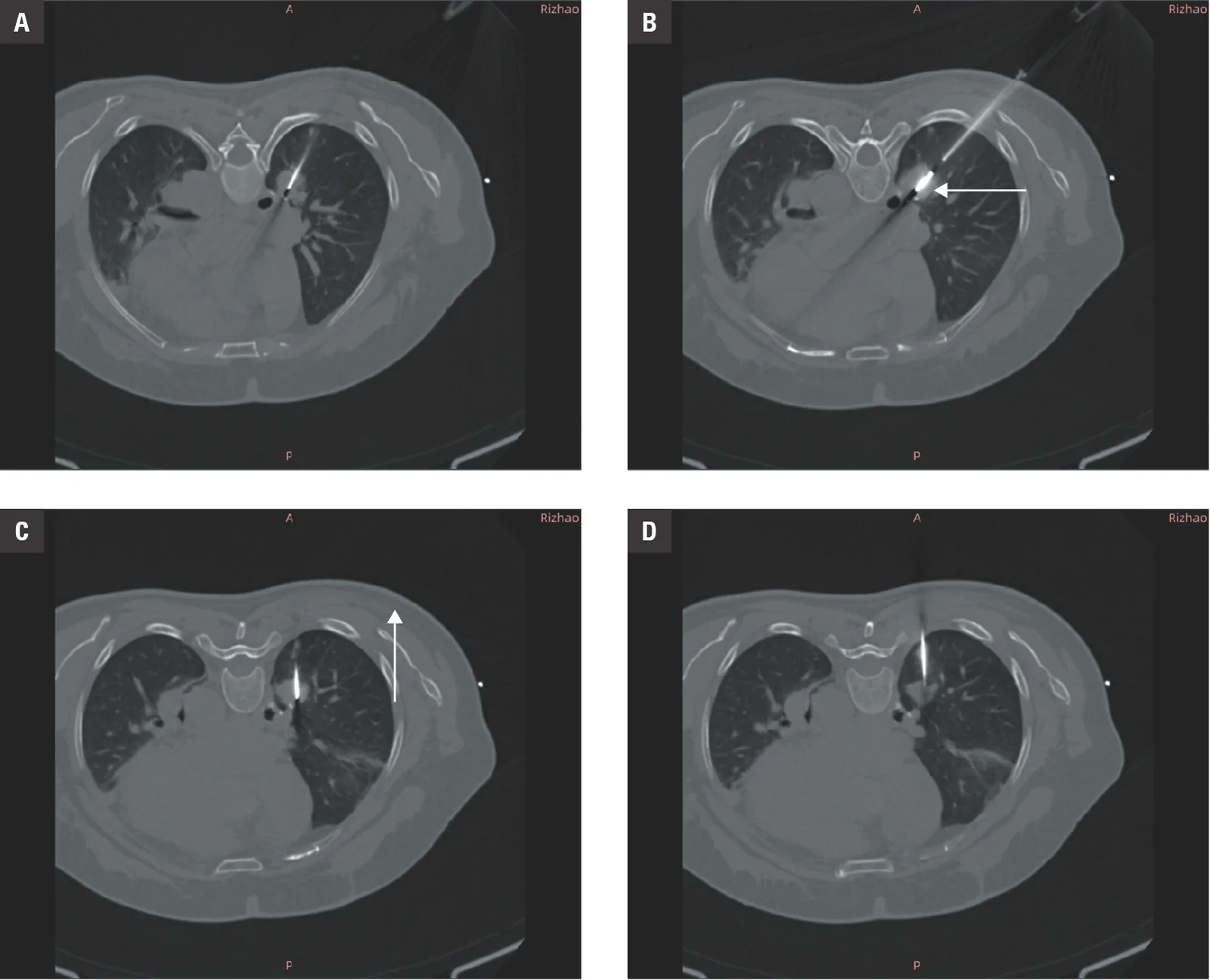
**A**–**D** – chest computed tomography performed during microwave ablation (MWA) combined with particle implantation showing insertion of the MWA needle (**B**, arrow) and the particle implantation needle (**C**, arrow) into the target location, according to the preoperative plan

**FIGURE 3:**
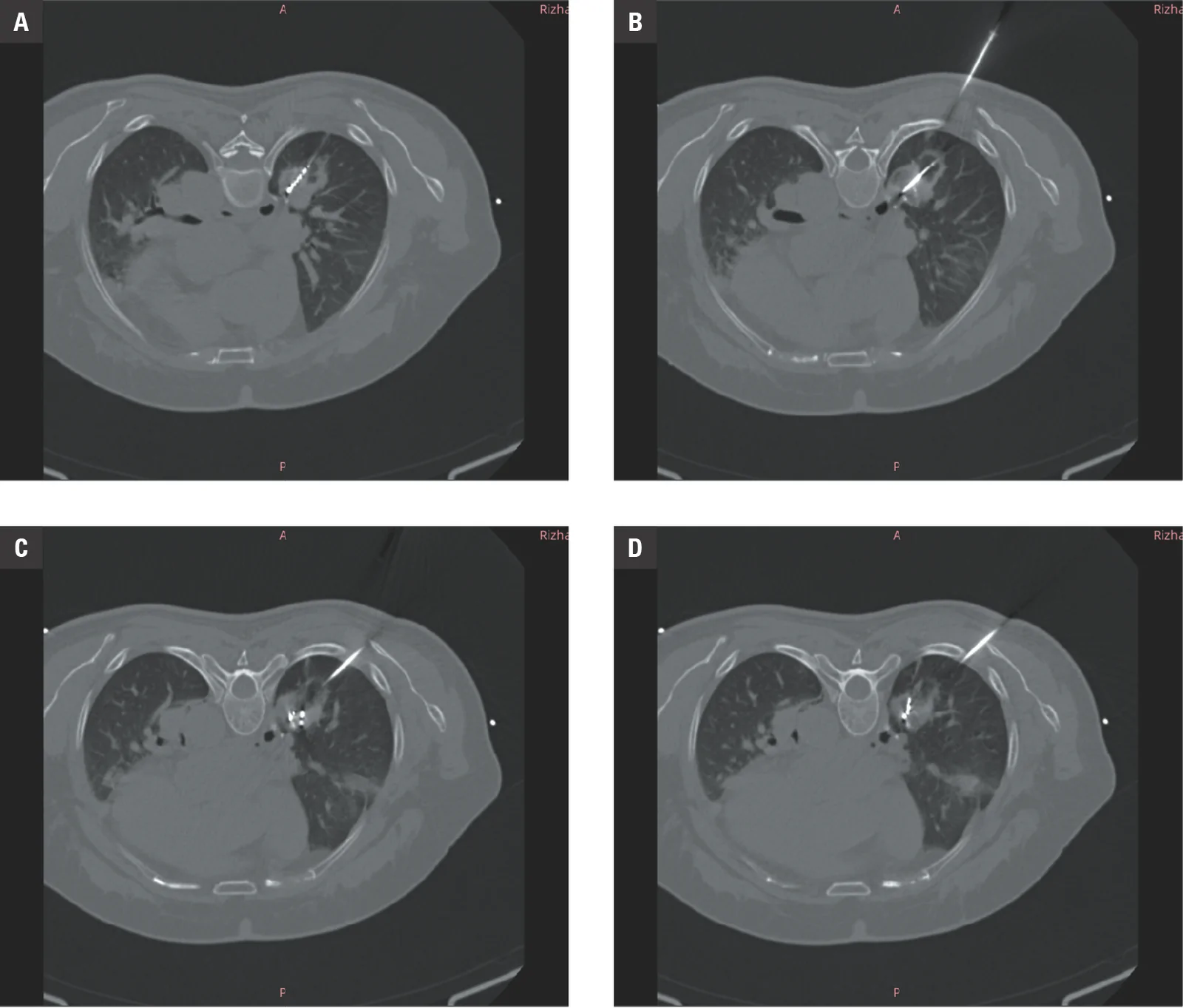
**A**–**D** – chest computed tomography performed during microwave ablation combined with particle implantation to assess the microwave ablation range, radioactive particle placement, and occurrence of complications

**FIGURE 4:**
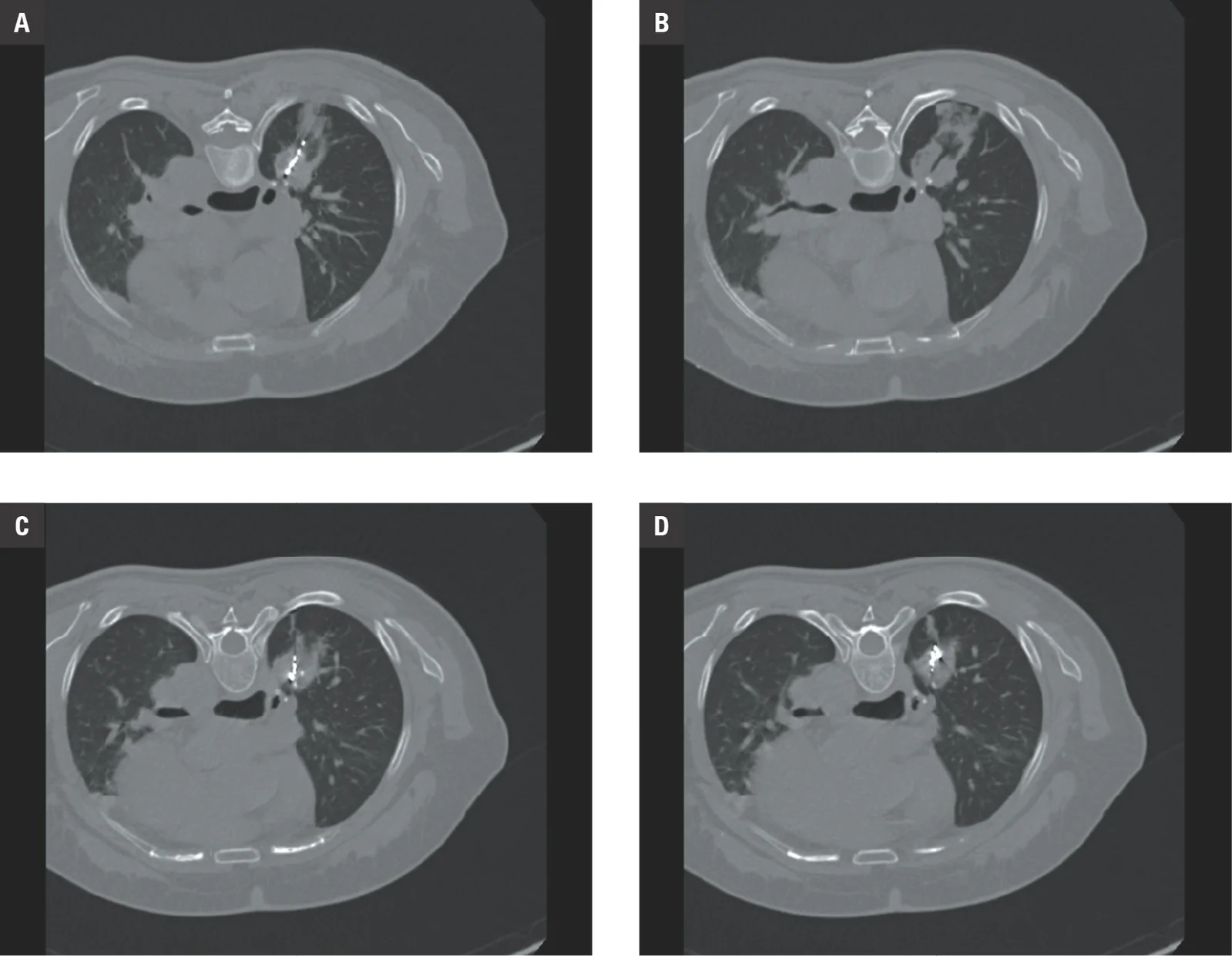
**A**–**D** – chest computed tomography performed 10 minutes after microwave ablation combined with particle implantation, showing a satisfactory ablation area, uniform particle implantation distribution, and minimal bleeding from the needle tract

The technical success rate was 100% in both groups. At the 3-month postoperative follow-up, the total effective rate was 70% (21/30) and the local control rate was 100% (30/30) in the control group, compared with 75% (21/28) and 100% (28/28), respectively, in the experimental group. No differences were observed between the groups in terms of the total effective rate and local control rate (Z = –0.422; *P* = 0.67). Similarly, at the 6-month postoperative follow-up, the total effective rate in the control group was 80% (24/30) and the local control rate was 100% (30/30), versus 82.1% (23/28) and 100% (28/28), respectively, in the experimental group. Again, no differences were noted between the groups in the total effective rate and local control rate (Z = –0.206; *P* = 0.84). Detailed data are presented in TABLE 3. Imaging data of a patient who underwent simultaneous MWA and particle implantation are presented in FIGURES 1–4.

**TABLE 3:** Comparison of follow-up data in the 2 groups

Follow-up time point	Control group (n = 30)	Experimental group (n = 28)	*P* value
One month			
CR	16 (53.3)	17 (60.7)	0.61
PR	8 (26.7)	6 (21.4)	
SD	6 (20)	5 (17.9)	
PD	0	0	
Three months			
CR	25 (83.3)	23 (82.1)	0.67
PR	5 (16.7)	5 (17.9)	
SD	0	0	
PD	0	0	
Six months			
CR	28 (93.3)	27 (96.4)	0.84
PR	2 (6.7)	1 (3.6)	
SD	0	0	
PD	0	0	

Data are presented as number (percentage) of patients.

Abbreviations: CR, complete response; PD, progressive disease; PR, partial response; SD, stable disease

## DISCUSSION

With the growing prevalence of low-dose computed tomography (LDCT) and advancements in artificial intelligence, the detection rate of lung nodules has surged, demonstrating a tendency toward multiple occurrences among younger individuals.^8^ An LDCT scanning scheme is used to reduce patient radiation exposure.^9^ Thoracoscopic wedge resection or segmentectomy can accomplish complete removal of the lesion while enabling pathological biopsy, achieving a diagnostic accuracy rate of 100%. Consequently, numerous scholars advocate for the benefits of resection surgery.^10^ However, for nodules located near the lung hilum or in other challenging locations, surgical resection may cause significant trauma, resulting in substantial loss of lung tissue and compromised lung function. Postoperative complications can also harm patients, as early surgical intervention does not substantially improve the overall patient survival rate.^11^^,^^12^

Thermal ablation has continuously progressed in tumor therapy, playing a pivotal role in the treatment of patients with advanced lung cancer or metastatic lung tumors with no surgical indications.^13^ MWA typically utilizes 2 frequencies: 915 MHz and 2450 MHz. Under the influence of microwave electromagnetic fields, polar molecules, such as water and protein molecules in tumor tissue, undergo extremely rapid vibrations, causing mutual collisions and friction. This leads to a rapid increase in temperature up to 60–150°C, resulting in cellular coagulation necrosis. The microwave energy is focused within a specific area by an antenna, enabling effective radiation to the target area. Notably, microwave thermal radiation exhibits a higher convection coefficient and a lesser heat deposition effect in the lungs.^14^ Literature reports suggest that the complete ablation rate of tumors located in the outer one-third of the lung is remarkably higher than that of tumors in the middle and inner one-third.^15^ This is attributed to the proximity of the middle and inner parts of the lungs to the heart, large blood vessels, and main airways. In consideration of these factors, it is worth exploring whether the use of combined methods can enhance efficacy of the procedure. Studies have found that ^125^I particle irradiation activates the p38MAPK/MDM2/p53 signaling pathway, promoting apoptosis in non-small cell lung cancer (NSCLC) cells.^16^ Relevant guidelines indicate that when the tumor diameter exceeds 5 cm or its location may impact the ablation effect, combined treatment with alternative methods can be considered.^17^ ^125^I seed implantation, a form of internal radiation therapy, has proved effective in the treatment of prostate cancer,^18^ brain tumors,^19^ and recurrent cervical cancer.^20^ As a form of local brachytherapy, after being introduced and continuously refined by Wang et al,^21^ Jiang et al,^22^ and Qu et al,^23^ combined with various auxiliary guidance techniques, this treatment approach is now extensively employed in the clinical management of solid malignant tumors in various locations. Yu et al^24^ reported that ^125^I seed implantation exhibited superior effectiveness in treating stage III recurrent NSCLC patients, with a prolonged median survival time and no severe complications. In our research, the average length of hospital stay in the experimental group was shorter than that in the control group, although no significant difference in efficacy and treatment efficiency was observed between the 2 groups. This indicates that the use of ^125^I seed implantation for close-range radiotherapy in conjunction with MWA for pulmonary nodules may shorten patients’ hospitalization and potentially reduce their economic burden to a certain extent.

Primary complications associated with MWA and particle implantation include pneumothorax, hemorrhage, and pleural effusion. These complications are influenced by factors such as the lesion’s location, size, depth, and elasticity, as well as the compliance of the lung tissue.^25^ Here, longer localization time was identified as a risk factor significantly associated with the odds of pneumothorax and lung hemorrhage, whereas positional change was specifically associated with the risk of pneumothorax.^26^ When MWA targets pulmonary nodules situated in the proximity to the pleura, pericardium, and lung hilum, it may lead to severe complications such as pleural fistulas, nerve injuries, and substantial bleeding, potentially impairing patients’ quality of life and even posing life-threatening risks. Consequently, this study aimed to address high-risk pulmonary nodules by integrating MWA with particle implantation, in order to achieve complete remission.

The results demonstrated that the technical success rate for CT-guided MWA, combined with either simultaneous or delayed particle implantation, in treating pulmonary nodules was 100%. The treatment efficacy for nodules, assessed at 3- and 6-month follow-up, exceeded 70%, fully aligning with clinical expectations. No differences were observed between the 2 groups in terms of the incidence of pleural effusion, hemoptysis, and pneumothorax, as well as the overall effective rate of nodule control. These findings suggest that both methods are comparably effective and safe. However, patients undergoing MWA combined with simultaneous particle implantation had a shorter total hospital stay compared with those treated with MWA combined with delayed particle implantation.

## CONCLUSIONS

This study shows that MWA in combination with particle implantation offers high safety and a reduced incidence of complications. By ensuring surgical efficacy without augmenting complication rates, this approach can shorten patients’ hospitalization, alleviate their economic burden, and enhance their overall medical experience. It can be successfully used to treat lung nodules with minimal trauma, precise short-term effectiveness, and limited harm. However, the study is limited to cases of multiple lung nodules located in the same lung lobe, insufficient samples, and a relatively short follow-up period. Consequently, long-term clinical efficacy assessment requires additional follow-up, which may inherently introduce occasional inaccuracies. Further research grounded in extensive clinical big data is required to establish whether this combined therapy can supersede surgery as the primary treatment for lung nodules.
